# Minimal One-Third Incision and Four-Step (MOTIF) Excision Method for Lipoma

**DOI:** 10.1155/2021/4331250

**Published:** 2021-08-26

**Authors:** Joseph Kyu-hyung Park, Jinhyun Kim, Jong-Ho Kim, Seokchan Eun

**Affiliations:** Department of Plastic and Reconstructive Surgery, Seoul National University College of Medicine, Seoul National University Bundang Hospital, Republic of Korea

## Abstract

Excision is the gold standard for lipomas. Patients desire minimal scars, but minimal incisions can increase complications and produce hypertrophic scars. We propose an algorithmic method named the minimal one-third incision and four-step extraction method (MOTIF) for lipoma excision. This retrospective study analyzed lipomas surgically excised using the MOTIF method at our institution between January 2016 and December 2018. A total of 112 lipomas were included. The complication rates and Vancouver Scar Scale (VSS) for three different size groups (<3 cm, 3 ~ 6 cm, >6 cm) were compared. Complete excision of all palpable lipomas was achieved with this approach. There were two seromas, two hematomas, and one postoperative nerve injury. There was no difference in complication rates and VSS between the three size groups. The MOTIF method is a cost-effective, reliable, and cosmetically pleasing method that can be applied to all lipomas regardless of size and location.

## 1. Introduction

Lipomas are the most common mesenchymal origin neoplasm, with a prevalence ranging from 0.1 to 3% [1, 2]. Most lipomas are slow-growing and small, therefore often left untreated. However, lipomas can grow into larger, even giant tumors, causing aesthetic and functional problems [2, 3]. In rapidly growing lipomas, radiologic evaluations followed by surgical intervention are necessary to differentiate the mass from malignant neoplasms such as liposarcomas [4].

Many treatment options are available, ranging from injection lipolysis and liposuction to simple and wide excisions. By allowing complete excision of the mass with a low recurrence rate, traditional surgical excision remains the treatment of choice [4]. Depending on the size of the mass, various surgical excision methods may be used. For small lipomas, punch biopsy, linear incision, and elliptical excision are available options [1, 5–7]. For larger lipomas, longer incision, large elliptical incision, or Z-incision designs have been advocated for a better surgical view [4, 7, 8].

Over the past two decades, minimally invasive procedures have become more popular among patients due to higher satisfaction. Patients desire shorter scars and less invasive procedures. However, for beginning surgeons, limited incisions are technically challenging. Furthermore, the limited incision may result in tissue injury and maceration due to unnecessary traction, which causes hypertrophic scar formation. Here, we present a simple and practical surgical approach, the minimal one-third incision and four-step (MOTIF) method for lipomas that allow a sufficient surgical field while producing comparable scars to traditional full-length incisions.

## 2. Materials and Methods

A retrospective chart review was performed on histologically confirmed lipomas excised by a single surgeon from January 2016 to December 2018. Operations were performed under either local anesthesia or monitored controlled anesthesia. Patient demographics, size and location of the mass, and complications were collected.

### 2.1. Operative Technique

A retrospective chart review was performed on histologically confirmed lipomas excised by a single surgeon from January 2016 to December 2018. Operations were performed under either local anesthesia or monitored controlled anesthesia. Patient demographics, size and location of the mass, and complications were collected.

The MOTIF technique is described as follows.

#### 2.1.1. Step I (Figure 1)

Mark the boundary of the lipoma with thorough palpation. Generously inject local anesthetic solution using 1% lidocaine with epinephrine (1 : 100,000). A minimum of five minutes is needed for adequate diffusion of epinephrine and minimize bleeding. Thorough hydro-dissection of both the superficial surface and the deep surface of the mass is crucial in maintaining a bloodless surgical field and allowing easy dissection of the mass. Minimal incision spanning one-third of the long axis is marked. If the incision along the long axis does not coincide with the relaxed skin tension line, the incision can be modified accordingly. The incision is made using a No. 15 scalpel blade. A minimum of 1 cm incision is needed for smaller masses to insert surgical equipment into the field. Dissection is continued until the superficial surface of the lipoma is exposed.

#### 2.1.2. Step II (Figure 2)

While holding a retractor (a skin hook followed by a Ragnell retractor) in the nondominant hand, dissection of the superficial surface is performed in a circumferential fashion using the dominant hand. Using Metzenbaum scissors or mosquito forceps is preferred over electrocautery to minimize thermal injury.

#### 2.1.3. Step III (Figure 3)

The next step is the dissection of the deep surface of the mass. Dissection should begin at the tip of the shorter axis of the mass. After the maximum dissection possible from this angle is performed, further dissection should be made on the opposite end of the shorter axis. Dissection of both ends of the shorter axis allows partial mobilization of the mass within the pocket. Next, the dissection should continue circumferentially until a stalk is left in the center of the mass.

#### 2.1.4. Step IV (Figure 4)

The mass is then squeezed through the opening to expose the stalk. The mass can now be easily removed after dissection of the stalk. After meticulous hemostasis and irrigation of the pocket, the wound is closed layer by layer. For masses larger than 2 cm in size, a silastic drain is utilized to minimize seroma formation.

### 2.2. The Complication and Vancouver Scar Scale Analysis

**Figure 5:**
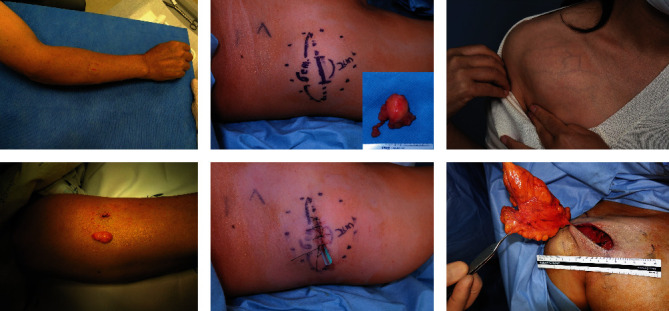
Three groups based on size: small (<3 cm, (a))—notice the minimum incision needed is about 1 cm; intermediate lipomas (3 ~ 6 cm, (b)); large lipomas (>6 cm, (c)).

A retrospective chart review was done to identify any postoperative complications such as seroma, hematoma, nerve injury, or recurrence. Scar quality was evaluated using the Vancouver Scar Scale (VSS) by a single plastic surgeon during outpatient clinic visits, a minimum of six months after the operation. To compare complication rates and VSS scores, all patients were divided into three groups based on the mass size: <3 cm, 3 ~ 6 cm, and >6 cm. For small masses, less than 3 cm, an incision of at least 1 cm was made to allow entry of surgical equipment. Therefore, lipomas less than 3 cm in diameter were grouped as “small” lipomas. Based on the senior surgeon's experience, lipomas larger than 6 cm required more careful dissection, meticulous hemostasis, and more traction through smaller incisions; lipomas larger than 6 cm were grouped as “large” lipomas and were at higher risk of postoperative complications. Lipomas between these two ranges were grouped as “intermediate” lipomas (Figure 5).

### 2.3. Statistical Analysis

A one-way ANOVA test was used to compare complication rates and VSS scores between different size groups. A *p* value < 0.05 was considered statistically significant, and all statistical analyses were done using RStudio (Boston, Mass.) and Microsoft Excel (Microsoft Corp., Redmond, Wash.).

## 3. Results and Discussion

### 3.1. Results

A total of 112 patients (129 lipomas) underwent lipoma excision using the MOTIF method. The mean age was 46 years old (20-82 years old), and 47 males and 65 females were included. Lipomas were most common in the trunk (40.3%), followed by the extremities (26.4%), the neck (17.1%), the scalp (10.1%), and the face (6.2%). The mean size was 5.98 cm (0.7–25 cm). When divided into the three size groups described above, 15.5% of the masses were less than 3 cm in size, 54.2% were between 3 and 6 cm, and 30.2% were larger than 6 cm (Table 1).

There were 2 cases of seroma (1.6%), two hematomas (1.6%), and one nerve injury (0.8%). Seromas were treated with aspiration, and hematomas were evacuated under local anesthesia. There were one, one, and three complications for small, intermediate, and large size groups, respectively. There was an increasing trend of complications in the large group. However, one-way ANOVA showed no statistical difference in complication rates between the three size groups (*p* = 0.144) (Table 2).

**Figure 6:**
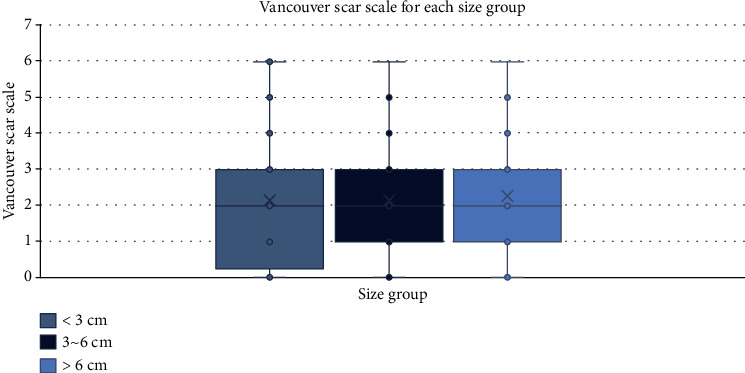
Comparison of the total Vancouver Scar Scale between the three size groups (one-way ANOVA, *p* = 0.81).

The mean VSS was 2.17 (*σ* = 1.51, range 0-6) for the entire sample, and the scores were 2.15, 2.14, and 2.23 for small, intermediate, and large size groups. One-way ANOVA showed no statistical difference between the three groups (*p* value = 0.81, Figure 6).

## 4. Discussion

More patients are seeking minimally invasive operations in hopes of reducing visible scars and deformities. However, “minimally invasive” does not necessarily mean that a patient will tolerate the increased risk of complications or suboptimal results. Direct excision has been the treatment of choice for lipomas. Several methods such as lipolysis, liposuction, minimal excision, and endoscopic excision have been used to reduce scars [4]. Liposuction and lipolysis produce minimal scars. However, liposuction does not allow the surgeon to visualize the tumor and often causes lipoma fragmentation. Moreover, seeding through cannula tracks [6] might increase the risk of local recurrence if the final pathology reports a malignant tumor.

Larger or giant lipomas often require extensive dissection, more meticulous hemostasis, and extended surgical time [5]. Long incisions or elliptical incisions have been advocated for a better surgical view. More complicated designs such as Z-plasty incisions have also been proposed [8]. However, initial Z or half Z incisions have the drawback of sacrificing the option of extending the incision middissection if circumferential dissection through the limited incision is impossible due to severe adhesion.

Incision lengths reported in the literature have a large spectrum ranging from as small as 30% to 100% of the longest axis of the mass [9–11]. Limited incisions have the possibility of increased complications and formation of hypertrophic scars due to excessive traction. In our study population, all lipomas were successful using the MOTIF method.

To evaluate the risk of complications, patients were divided into three groups based on the mass size as described above. Complication rates between small, intermediate, and large groups did not exhibit statistically significant differences, but the overall complication rate was slightly higher in the large group. In addition, there was one case of nerve injury causing paresthesia of the lower back in the large size group, which resolved spontaneously after one year. Seroma formation is associated with thermal injury [12]. The use of Metzenbaum scissors and mosquito forceps for dissection rather than electrocautery and the use of bipolar electrocautery for hemostasis have the advantage of reduced seroma formation in large lipomas.

VSS scores were comparable between the three size groups. Larger lipomas often require stronger traction for adequate surgical view. Two tips of the MOTIFS method can prevent excessive traction.

**Figure 7:**
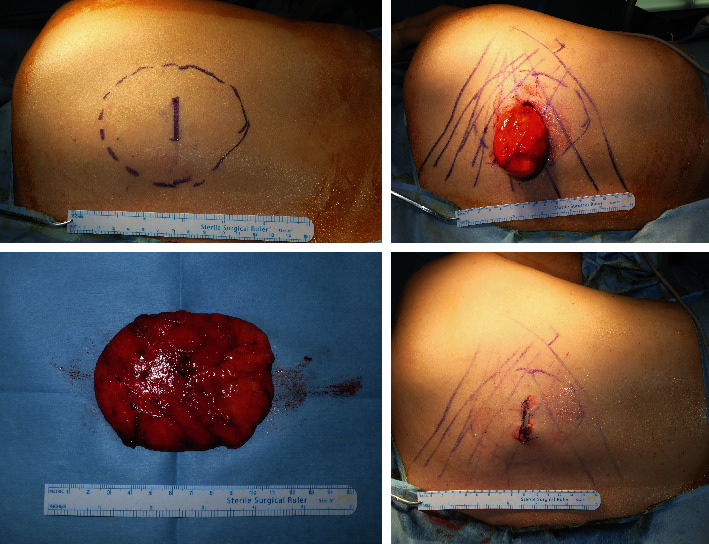
A 37-year old female patient with subcutaneous lipoma of the right upper back. Using the MOTIF method, 9.8 cm lipoma was excised. No complication such as hematoma, seroma, nerve injury, or recurrence was observed during the 6 months follow-up ((a) preoperative MOTIF design, (b) “Step IV” where the lipoma is delivered through the incision with the stalk attached, (c) excised lipoma, (d) postoperative photograph).

First, judicious local anesthetic solution injection and sufficient waiting time after injection are essential during “Step I.” Injection until a strong skin turgor is achieved, similar to tumescent solution injection for liposuction, allows hydro-dissection of the mass and the surrounding tissue. Increased interstitial pressure makes dissection easier. Additional injection underneath the mass can be made in “Step IV” for easier delivery of the mass through the incision [11]. For large lipomas, the local injection solution can be further diluted with normal saline to reduce lidocaine and epinephrine toxicity (Figure 7).

The second tip is a complete dissection of the mass' superficial margin and circumferential axial margin before approaching the underside of the mass. All fibrous attachments, if any, should be released, allowing full mobilization of the mass in all directions, minimizing traction injury. Lastly, dissection of the stalk after delivery of the mass through the incision further minimizes traction injury.

There are several limitations of this study. Long-term follow-up data were not available. Lipomas are slow-growing, and recurrence often goes unnoticed by the patients. Due to the benign nature of lipomas, it is difficult to follow-up with the patients for long periods. Further study of direct comparison between the MOTIF method and other minimally invasive methods such as liposuction and minimal incision methods is needed.

## 5. Conclusions

The MOTIF method is a systematic approach to lipomas of all sizes with low complication rates and satisfactory scar results. For beginning surgeons, remembering the four stages will be useful in safe excisions using small incisions, even for large lipomas.

## Figures and Tables

**Figure 1:**
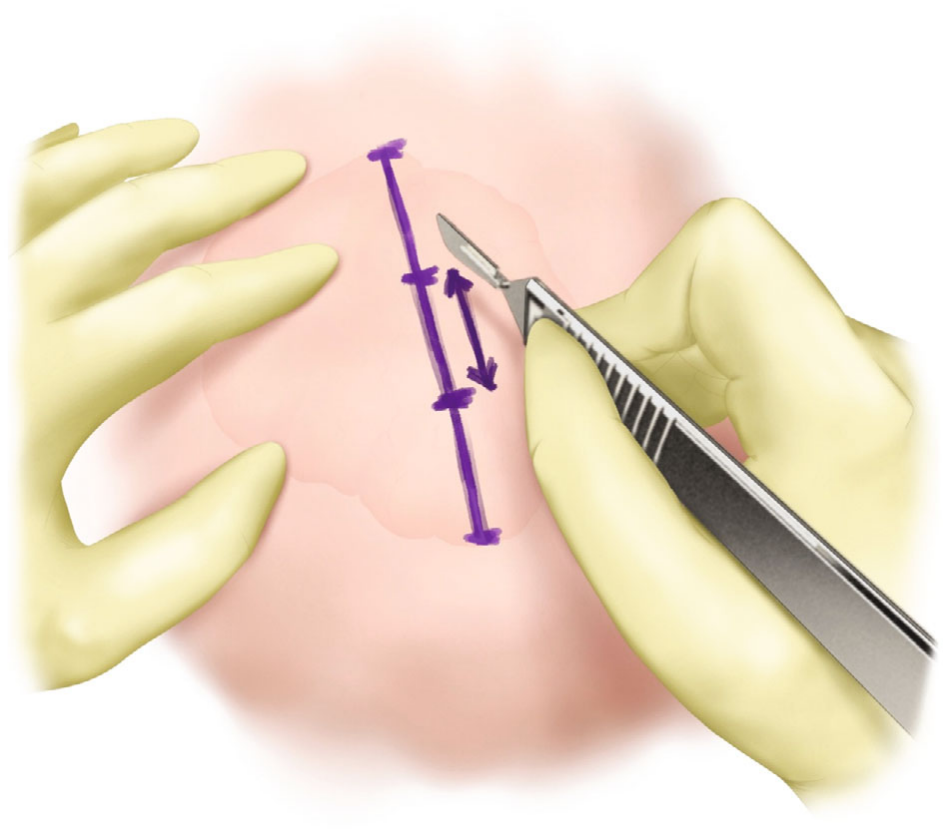
Step I of MOTIF. The boundary of the lipoma is marked. Minimal incision spanning one-third of the long axis is marked. The incision is made after ample local anesthetic injection and waiting a minimum of five minutes for vasoconstriction.

**Figure 2:**
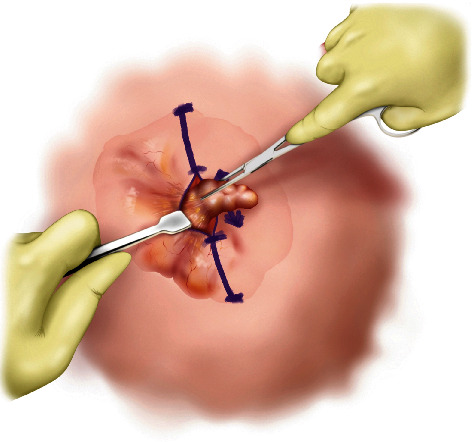
Step II of MOTIF. While holding a retractor in the nondominant hand, dissection of the superficial surface performed circumferentially.

**Figure 3:**
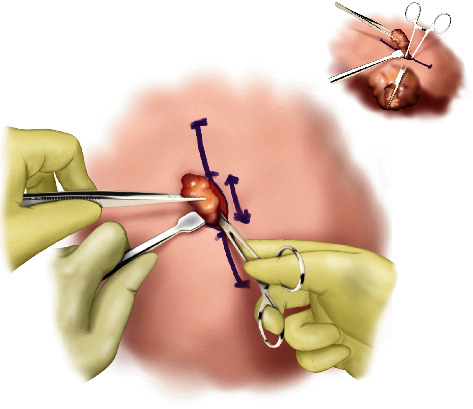
Step III of MOTIF. Dissection of the deep surface is performed starting with the shorter axis of the mass for easier mobilization. Circumferential dissection is performed until a stalk is left in the center of the mass.

**Figure 4:**
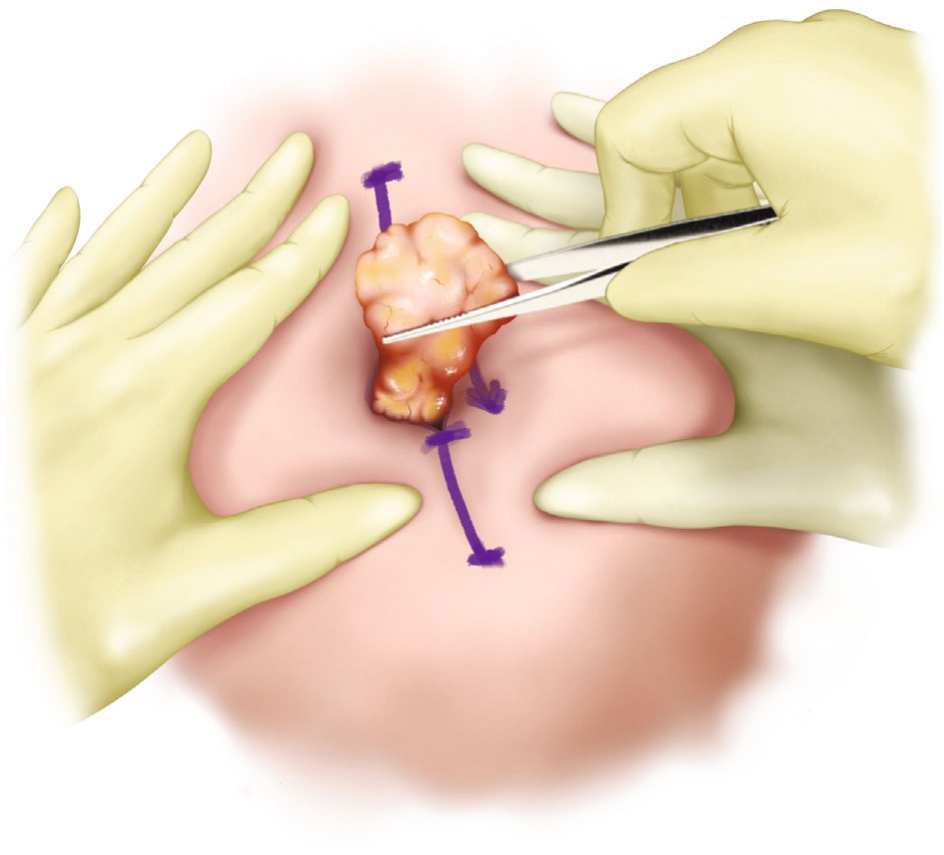
Step IV of MOTIF. The mass is squeezed through the opening, and the stalk is cut. After meticulous hemostasis and irrigation, the wound is closed layer by layer.

**Table 1:** Patient demographics.

Patient demographics	
Variable	Value
No. of patients	112
Mean age ± SD (range), yr	46.28 ± 10.1 (20-82)
Sex	
Male	47
Female	65
Location	
Trunk	52 (40.3%)
Extremities	34 (26.4%)
Neck	22 (17.1%)
Scalp	13 (10.1%)
Face	8 (6.2%)
Mean size ± SD (range), cm	5.98 + 4.76 (0.7-25)
Size	
<3 cm	20 (15.5%)
3 ~ 6 cm	70 (54.2%)
>6 cm	39 (30.2%)
Mean follow − up ± SD (range), mo	8.28 ± 4.6 (3-38)

**Table 2:** Complications of MOTIF.

Complication	<3 cm	3 ~ 6 cm	>6 cm	Total
Seroma	0	1 (1.4%)	1 (2.6%)	2 (1.6%)
Hematoma	1 (5.0%)	0	1 (2.6%)	2 (1.6%)
Nerve injury	0	0	1 (2.6%)	1 (0.8%)
Recurrence	0	0	0	0
			*p* = 0.144	

## Data Availability

Data of this study are available upon request.
